# Rehabilitation after ACL Injury: A Fluoroscopic Study on the Effects of Type of Exercise on the Knee Sagittal Plane Arthrokinematics

**DOI:** 10.1155/2013/248525

**Published:** 2013-08-26

**Authors:** Sadegh Norouzi, Fateme Esfandiarpour, Ali Shakourirad, Reza Salehi, Mohammad Akbar, Farzam Farahmand

**Affiliations:** ^1^Physical Therapy Department, School of Rehabilitation, Ahvaz Jundishapur University of Medical Sciences, Ahvaz 61357-15794, Iran; ^2^Musculoskeletal Rehabilitation Research Center, Ahvaz Jundishapur University of Medical Sciences, Ahvaz 61357-15794, Iran; ^3^Advanced Diagnostic and Interventional Radiology Research Center, Tehran University of Medical Sciences, Tehran 14197-33141, Iran; ^4^School of Mechanical Engineering, Sharif University of Technology, Tehran 11155, Iran; ^5^RCSTIM, Tehran University of Medical Sciences, Tehran 14197-33141, Iran

## Abstract

A safe rehabilitation exercise for anterior cruciate ligament (ACL) injuries needs to be compatible with the normal knee arthrokinematics to avoid abnormal loading on the joint structures. The objective of this study was to measure the amount of the anterior tibial translation (ATT) of the ACL-deficient knees during selective open and closed kinetic chain exercises. The intact and injured knees of fourteen male subjects with unilateral ACL injury were imaged using uniplanar fluoroscopy, while the subjects performed forward lunge and unloaded/loaded open kinetic knee extension exercises. The ATTs were measured from fluoroscopic images, as the distance between the tibial and femoral reference points, at seven knee flexion angles, from 0° to 90°. No significant differences were found between the ATTs of the ACL-deficient and intact knees at all flexion angles during forward lunge and unloaded open kinetic knee extension (P < 0.05). During loaded open kinetic knee extension, however, the ATTs of the ACL deficient knees were significantly larger than those of the intact knees at 0° (*P* = 0.002) and 15° (*P* = 0.012). It was suggested that the forward lunge, as a weight-bearing closed kinetic chain exercise, provides a safer approach for developing muscle strength and functional stability in rehabilitation program of ACL-deficient knees, in comparison with open kinetic knee extension exercise.

## 1. Introduction

Anterior cruciate ligament (ACL) injuries are reported to be the most common knee ligament injury, with an estimated rate of 1 per 3,000 in general population [1]. A rehabilitation program is an essential and integral part of treatment after ACL injury, with the objective of promoting the muscular strength and reestablishing the knee joint functional stability [2–4]. The rehabilitative exercises need to be compatible with normal arthrokinematics to avoid abnormal stresses on the tibiofemoral joint articulating surfaces and to protect other joint structures from overloading [3].

The kinematics behavior of the knee during common rehabilitation exercises, that is, lunge [5], squat, leg press [6, 7], step up [8], open kinetic knee extension [4, 7], and straight leg raising [9] has been the focus of several studies in the literature, in search for safer rehabilitation procedures. In general, closed kinetic chain (CKC) exercises are suggested to provide improved arthrokinematics in comparison with open kinetic chain (OKC) exercises for rehabilitation of ACL injury [7, 10, 11], due to the muscular cocontraction, as well as the weight bearing and the resulting joint compressive forces [12–14]. This suggestion is based on the observation that CKC exercises produce a smaller magnitude of anterior tibial translation (ATT) than OKC activities [7, 15]. However, clinical studies have often failed to provide sufficient scientific evidence to support the superiority of CKC exercises in terms of functional outcomes, subjective symptoms, and knee stability [16, 17]. Some investigations [15, 18, 19] have reported that the kinematics effects, resulting from hamstrings coactivation and increase of the joint compression force during CKC exercises, are not sufficient to reduce the ATT significantly. In fact, there are also reports of larger ATTs and similar ACL strains during CKC compared with OKC exercises [18, 20].

Apart from methodological inadequacies, the discrepancy of the results of previous studies might be attributed to the specific kinematic and dynamic conditions associated with each individual exercise. It has been reported that the knee joint kinematics, and particularly the ATT, is highly activity dependent and is affected by the level of the quadriceps activation and hamstring and gastrocnemius cocontraction, which might be quite different even among various CKC exercises [12, 21].

The objective of the present study was to measure the amount of ATT during forward lunge and unloaded/loaded open kinetic knee extension, which are among the most common CKC and OKC exercises, respectively. An improved methodology, based on landmark registration of fluoroscopic images, was used to measure the sagittal plane arthrokinematics of intact and ACL-deficient knees throughout a functional range of motion during exercises. The results were then used to provide suggestions for physical therapists to prescribe safer rehabilitation programs for ACL-deficient patients.

## 2. Materials and Method

Fourteen male volunteers (mean age = 35.8 ± 9.5), suffering from complete unilateral ACL rupture, participated in the study. The sample size was determined by priori sample-size power analysis (*β* = 0.20 and *α* = 0.5), based on the preliminary results of a pilot study on four subjects. The sex of the participants was considered to be the same, to reduce the gender bias, as recommended by the National Athletic Trainers' Association (NATA) for ACL injury research [22]. Selection of gender was based on the higher availability of male volunteer patients in our collaborating clinics. The ACL injury of subjects was documented via MRI and clinical examination of ACL's functionality, that is, positive Lachman, pivot shift, and anterior drawer tests, performed by an expert orthopedic surgeon. All subjects had received eight to twelve sessions of routine physiotherapy and were in the waiting list for ACL reconstruction surgery.

The volunteers were reexamined by an expert orthopedic surgeon for inclusion/exclusion criteria before participating in the tests. Subjects were excluded if they had any other associated injuries, pain during testing, more than a trace effusion, restriction of motion in hip, knee, and ankle joints, apparent skeletal mal alignments, such as genuvarum/valgum, determined via clinical examination [23], and any contraindications to X-ray imaging. The mean time interval between the occurrence of injury and the test of the participants was 9.1 (±2.1) months. All participants signed an informed consent approved by Human Investigations Committee of Ahvaz Jundishapur University of Medical Sciences, Iran.

A uniplanar fluoroscopy system (C-ARM DSP-A2000 model, Toshiba, Japan) was used to capture lateral fluoroscopy video data (12 frames per second) from the subjects' knees while they performed three exercises in a random order: (1) forward lunge, (2) unloaded open kinetic knee extension, and (3) loaded open kinetic knee extension against a 2 kg resistance, using a weighted cuff supported above the malleoli with Velcro. Selection of the 2 kg load was in consistency with previous studies in the literature [15, 18] and based on the results of our pilot study, indicating undue discomfort when using a 4 kg load. The forward lunge was performed from standing position to at least 90° knee flexion, with the test knee positioned forward, and then returning to the starting position. Subjects performed the movement at their own pace with no speed constraint in favor of functionality. For open kinetic knee extension, the subjects seated on a chair with their knees flexed at 90° and femurs at neutral rotational position and then performed a whole cycle of knee extension-flexion in unloaded or loaded conditions. A metal ball with known radius, securely attached to each subject's leg or thigh, was used to calibrate the image of each frame. Before performing each experiment, the subjects had a five-minute rest and practiced the following exercise for a couple of complete cycles.

**Figure 1:**
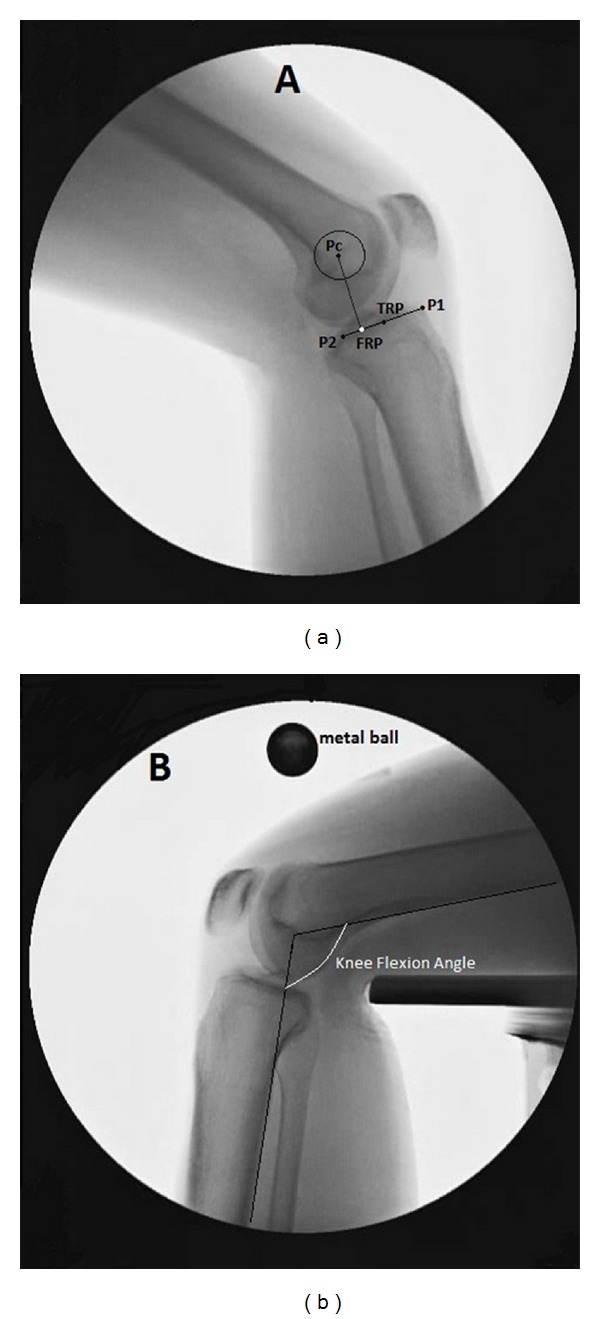
Analysis of the fluoroscopic images: (a) anatomical landmarks used in the study: (P1) anterior limit of the tibial plateau; (P2) posterior limit of the tibial plateau; (Pc) center of the best circle fitted to the posterior margin of the femoral inter-condylar notch. TRP: tibial reference point. FRP: femoral reference point. (b) the knee flexion angle was defined based on the femoral and tibial posterior axes. The metal ball used for magnification correction of images is also shown in the picture.

The fluoroscopy videos were decomposed into original frames using MATLAB software (version 7.10.0, MathWorks Inc., Natick, MA, USA). For the sake of consistency, only the frames related to the knee extension phases of motion, including the concentric (up) phase of the lunge maneuver, were used for analysis. The image of each frame was exported to the AutoCAD environment (ver. 2013, Autodesk Inc., Montreal, QC, Canada) for analysis (Figure 1(a)). The angle between the two lines, tangent to the posterior cortexes of the femoral and tibial shafts, was measured as the flexion angle [24, 25]. The extent of ATT relative to the femur was determined for both the intact (considered as control) and ACL-deficient knees, at seven knee flexion angles, from 0° to 90°, with 15° intervals. The ATTs were obtained based on registration of anatomical landmarks in successive image frames (Figure 1(b)) [26].

Three anatomical landmarks were used in our study, including the anterior limit of the tibial plateau (P1), the posterior limit of the tibial plateau (P2), and the center of the best circle fitted to the posterior margin of the femoral intercondylar notch (Pc) (Figure 1). These landmarks were manually digitized on the fluoroscopy image frames. A line was drawn to connect P1 and P2, and its midpoint was considered as the tibial reference point (TRF). Another line was drawn from Pc, perpendicular to the line P1-P2, with the intersection point considered as the femoral reference point (FRP). The ATT was determined in each image frame as the distance between the femoral and the tibial reference points. 

The interobserver and intraobserver reliability of the identification procedure of anatomical landmarks were tested by two trained observers, repeating the measurement process on two sessions two days apart. Interclass correlation coefficient was used to determine the reliability of landmark identification.

Multifactorial ANOVA statistical analysis was used to evaluate the dependence of the amount of ATT on the following measures: (1) knee condition at two levels (ACL-deficient, intact), (2) flexion angle at 7 levels (0°, 15°, 30°, 45°, 60°, 75°, and 90° of knee flexion), and (3) exercise at 3 levels (forward lunge, unloaded open kinetic knee extension, and loaded open kinetic knee extension). Post hoc testing with paired *t*-tests was used to evaluate the sources of main effects. Significance level was set at *α* < 0.05.

## 3. Results

The ICC for intraobserver and interobserver reliability of landmark identification were 0.93 and 0.89, respectively. The ATT increased with progressive knee flexion in both the intact and ACL-deficient knees for all three exercises. No significant interaction was observed between the knee condition and the flexion angle, the exercise and the flexion angle, and the knee condition and the exercise. The main effect was significant for the knee condition (*P* = 0.023), the exercise (*P* = 0.001), and the knee flexion angle (*P* = 0.001).

**Figure 2:**
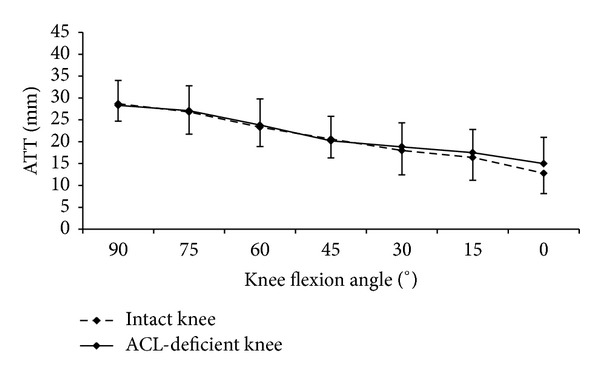
The anterior tibial translations (ATTs) of the ACL-deficient (ACLD) and intact knees against the knee flexion angle during forward lunge exercise.

**Figure 3:**
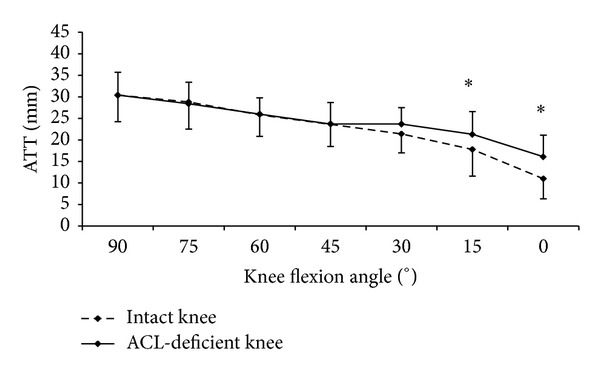
The anterior tibial translations (ATTs) of the ACL-deficient and intact knees against the knee flexion angle during loaded open kinetic knee extension. *Significant difference (*P* < 0.05).

**Figure 4:**
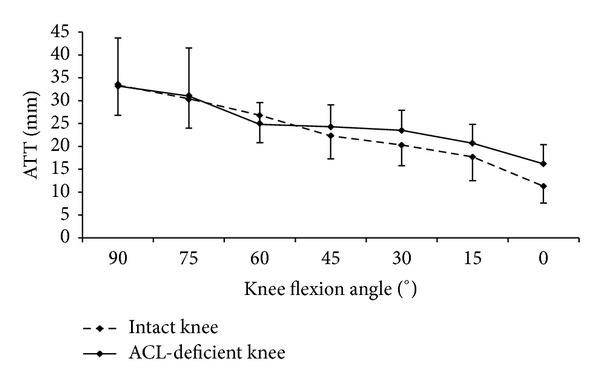
The anterior tibial translations (ATTs) of the ACL-deficient and intact knees against the knee flexion angle during unloaded open kinetic knee extension.

Comparing the ATTs of the intact and the ACL-deficient knees, no significant difference was found during the forward lunge at all flexion angles examined (Figure 2). For the loaded open kinetic knee extension, the ATTs of the ACL-deficient knees were significantly larger than those of the intact knees at 0° (15.5 ± 6 versus 10.9 ± 4.7, *P* = 0.002) and 15° (21.3 ± 5.3 versus 17.8 ± 6.2, *P* = 0.012) knee flexion, but not at 30° (Figure 3). For the unloaded open kinetic knee extension, the ATTs of the ACL-deficient knees were larger than those of the intact knees at 0°, 15°, and 30° knee flexion; however, the differences were not statistically significant (Figure 4). In general, the ATTs of the ACL-deficient knees at the end range of knee extension (30° knee flexion to full extension) were larger than those of the intact knees during all three exercises (Figures 2, 3, and 4). However, the differences were only significant for loaded open kinetic knee extension.

**Figure 5:**
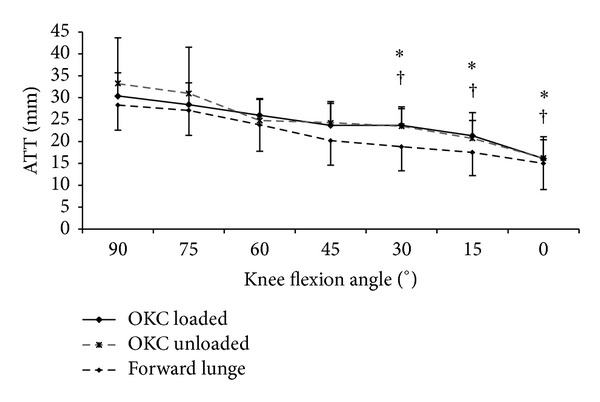
Comparison of the anterior tibial translations (ATTs) of ACL-deficient knees during forward lunge and unloaded and loaded open kinetic knee (OKC) extension. *Significant difference (*P* < 0.05) between forward lunge and OKC loaded extension. ^†^Significant difference (*P* < 0.05) between forward lunge and OKC unloaded extension.

For the ACL-deficient knees, the smallest ATTs were observed during the forward lunge exercise. The average of the ATTs over the range of knee flexion (between 0° and 90°) was 21.5 (±7.2) mm for forward lunge exercise, which was significantly smaller than that for loaded knee extension (24.2 ± 6.3, *P* = 0.001) and unloaded knee extension (24.8 ± 8.4, *P* = 0.001) (Figure 5). In a more detailed analysis, the ATTs of the ACL-deficient knees during forward lunge were significantly smaller than those during loaded knee extension at 15° (17.5 ± 5.3 versus 21.3 ± 5.3, *P* = 0.001), 30° (18.7 ± 5.5 versus 23.7 ± 3.8, *P* = 0.005), and 45° (20.2 ± 5.6 versus 23.7 ± 5, *P* = 0.034) knee flexion. Similarly, significantly smaller ATTs were found for the ACL-deficient knees during forward lunge in comparison with unloaded knee extension at 15° (17.5 ± 5.3 versus 20.7 ± 4.1, *P* = 0.015), 30° (18.7 ± 5.5 versus 23.5 ± 4.4, *P* = 0.014), and 45° (20.2 ± 5.6 versus 24.3 ± 4.8, *P* = 0.034) knee flexion. No significant difference was found between the ATTs of the ACL-deficient knees during loaded and unloaded knee extension at any flexion angle (Figure 5).

**Figure 6:**
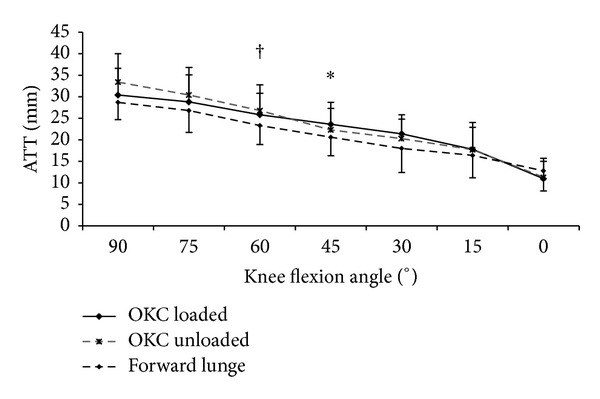
Comparison of the anterior tibial translations (ATTs) of intact knees during forward lunge and unloaded and loaded open kinetic knee (OKC) extension. *Significant difference (*P* < 0.05) between forward lunge and OKC loaded extension. ^†^Significant difference (*P* < 0.05) between forward lunge and OKC unloaded extension.

For the intact knees, the amount of ATTs during forward lunge was significantly smaller than those during loaded knee extension at 45° (20.6 ± 4.3 versus 23.6 ± 5.1, *P* = 0.040). A significantly smaller ATT was also found during the forward lunge in comparison with unloaded knee extension at 60° knee flexion (23.3 ± 4.4 versus 26.8 ± 6.0, *P* = 0.032.) (Figure 6). 

## 4. Discussion

The arthrokinematics of the ACL injured knees has been studied in previous investigations in a number of closed and open kinetic chain exercises [4, 6, 7, 15]. Also, there is an extensive clinical literature concerning the immediate effects [27–29] and the long-term outcome [17, 30, 31] of open and closed kinetic chain exercises of ACL rehabilitation. However, the results are often inconsistent which suggests the need for more accurate quantitative investigations to examine the safety and efficacy of each individual exercise.

Our results indicate that an open kinetic knee extension, especially against a resistive load, results in abnormally large anterior translations in ACL-deficient knees, at early knee flexion angles, that might impose abnormal loading to the joint structures. This was characterized with the significantly larger ATTs in ACL-deficient knees in comparison with the intact knees, during loaded open kinetic knee extension exercise, in the range of 30° knee flexion to full extension. Similar results have been reported by previous studies, indicating that in an open kinetic knee extension, increase of the external resistance leads to the increase of the ATT [18, 32], the ACL strain [20, 33–35], and the quadriceps activity level [15, 32, 36]. The results of a biomechanical modeling study by Mesfar and Shirazi-Adl [4] also suggest that application of a resistive load can significantly increase the ACL tension in a simulated open kinetic knee extension exercise.

For the forward lunge exercise, we found significantly smaller ATTs than open knee extension exercises for ACL-deficient knees, relatively similar to the results of the intact knees. This suggests that the ACL-deficient knees are stable against anterior-posterior translation during this closed chain weight-bearing exercise, with no need for a major restraining contribution from the ACL. Previous studies have often reported similar results, indicating significantly smaller ATTs during weight-bearing exercises compared to open kinetic knee extension [7, 8, 12, 37, 38]. However, there are also contradicting results for some closed kinetic chain exercises in the literature. For instance, Keays et al. [39] and Isaac et al. [18] reported larger or similar ATTs in ACL-deficient knees during wall squat and step-up activities, respectively, in comparison with open kinetic knee extension. Also, in a recent investigation, Esfandiarpour et al. [6] found a significantly larger ATT at 30° knee flexion in the ACL-deficient knees, in comparison with the intact knees, during leg press. We believe that this inconsistency of the kinematics behavior of the ACL-deficient knees during different close chain exercises might be attributed to the loading condition associated with each individual exercise, for example, the level of muscular cocontraction [40] and the magnitude of the joint compressive force [41].

In general, our results support the idea that forward lunge exercise, as a weight-bearing closed-chain exercise, provides a safer approach for developing muscle strength and functional stability in rehabilitation of ACL-deficient knees, in comparison with loaded open kinetic knee extension. This suggestion, however, is limited by the fact that our study only examined the knee joint arthrokinematics in sagittal plane. More sophisticated methodologies are required to be developed and employed in the future for dynamic assessment of the three-dimensional joint kinematics, using medical imaging modalities. 

Moreover, our results should not be considered a confirmation of the general guideline that CKC exercises are always safer than OKC activities. As it has been highlighted in our previous study [6], the knee arthrokinematics might be quite different during different CKC exercises. Therefore, before making any general conclusion, it is necessary to accurately measure and analyze the knee kinematics behavior for each specific exercise. Nevertheless, it should be noted that such kinematics investigations might only provide insight into the safety issues of a rehabilitation exercise. In order to explore the efficacy of an exercise, future studies should at the same time investigate the function of the muscle groups that are involved with the knee kinematics. Finally, in order to provide sufficient scientific evidence for supporting the safety and efficacy of an ACL rehabilitation program, more detailed clinical studies are needed to be performed in the future to characterize each individual exercise in terms of its subjective symptoms, immediate effects, and functional outcomes.

## 5. Conclusion

The aim of this study was to investigate the sagittal plane kinematics of intact and ACL-deficient knees during forward lunge and unloaded/loaded open kinetic knee extension. It was found that the anterior tibial translation of the ACL-deficient knees is similar to that of the intact knees during forward lunge, but different during open kinetic knee extension. This indicates that the level of muscular cocontraction and resulting joint compression during forward lunge provides the required sagittal plane knee stability in the absence of the ACL to restore the joint's normal arthrokinematics. Resistive knee extension in the range of 30° knee flexion to full extension, on the other hand, was found to be associated with abnormal joint kinematics that might impose abnormal loading to the joint structures. In general, our results support the idea that forward lunge exercise, as a weight-bearing closed-chain exercise, provides a safer approach for developing muscle strength and functional stability in rehabilitation of ACL-deficient knees, in comparison with loaded open kinetic knee extension.
